# Effects of a Doula instrument combined with auricular acupuncture and acupoint application on numerical rating scale scores, labour time of puerperae with a natural delivery and Apgar scores of the newborns

**DOI:** 10.12669/pjms.39.6.7451

**Published:** 2023

**Authors:** Bing Sun, Jing Ren, Ying Yang, Chunhong Yang

**Affiliations:** 1 Bing Sun, Department of Obstetrics, Baoding Maternal and Child Health, Hospital Baoding, 071000, Hebei, China; 2 Jing Ren, Department of Obstetrics, Baoding Maternal and Child Health, Hospital Baoding, 071000, Hebei, China; 3 Ying Yang, Department of Obstetrics, Baoding Maternal and Child Health, Hospital Baoding, 071000, Hebei, China; 4 Chunhong Yang, Department of Obstetrics, Baoding Maternal and Child Health, Hospital Baoding, 071000, Hebei, China

**Keywords:** Auricular acupuncture, Acupoint application, Doula instrument, Natural delivery, numerical rating scale, Labour time, Apgar score

## Abstract

**Objective:**

To determine how a combination of auricular acupuncture, acupoint application and the Doula instrument affects numerical rating scale (NRS) scores, labour time of puerperae and the Apgar scores of newborns during natural delivery.

**Methods:**

This is a retrospective study. From January 2021 to December 2022, clinical data were collected from 90 healthy primiparae who completed natural delivery at Baoding Maternal and Child Health Hospital. They were divided into two groups based on different perinatal intervention methods. While the Doula instrument was used for the control group’s perinatal intervention, the study group received a combination of auricular acupuncture, acupoint application and the Doula instrument during their perinatal period.

**Results:**

The NRS score of the study group was lower than that of the control group, demonstrating that their intergroup difference is statistically significant (*P* < 0.05). The incubation period, the active phase of the first stage of labour and the time of the second and third stages of work are shorter in the study group than in the control group (*P* < 0.05). Intergroup comparison of their one and five minutes Apgar scores demonstrates no statistically significant differences (*P* > 0.05). Expression levels in the study group are higher than in the control group five minutes after delivery (*P* < 0.05).

**Conclusions:**

In this case, a perinatal intervention comprising auricular acupuncture, acupoint application and the Doula instrument was used for puerperae undergoing natural delivery.

## INTRODUCTION

Delivery is a normal physiological process. Labour pain must be experienced by lying-in women throughout the delivery process. Labour pain stimulates the physiology and psyche of lying-in women, causing intense discomfort and endangering maternal and child health and safety.^1^ It is widely assumed that proper intervention in the delivery process is important. It can provide certain guarantees for the parturient and newborn safety.^2^ The Doula instrument is an effective intervention technique commonly used in the perinatal period. The Doula instrument affects the secretion of analgesic substances to achieve pain relief, which has produced outstanding outcomes and is safe for mothers and babies.^3^

According to the relevant literature, comprehensive perinatal interventions have the potential to provide even more outstanding results. At present, the Apgar score is often used to evaluate the physical conditions of newborns. Five major neonatal signs (i.e. skin colour, heartbeat, respiration, muscular tension and movement and reflex) are used to determine whether the newborns suffer from asphyxia and specific variations of asphyxia. In addition, TCM doctors have accumulated rich experience in perinatal interventions. Auricular acupuncture and acupoint application can produce precise effects that meet the delivery needs.^4^ On this basis, auricular acupuncture, acupoint application and a Doula instrument are combined in the present study to perform perinatal intervention for the parturient undergoing natural delivery.

## METHODS

This is a retrospective study. From January 2021 to December 2022, clinical data from 90 healthy primiparae who had a successful natural delivery at Baoding Maternal and Child Health were retrospectively collected and examined. Data were retrieved from the hospital information and management system. Various information of healthy primiparae were collected, including main clinical features. They were divided into two groups based on different perinatal intervention methods. Cases in the control group (n = 43) are 20-34 years old (mean age: 27.45 ± 3.11 years old) and 157-170 cm tall (mean stature: 164.07 ± 2.73 cm); their body masses range from 54 kg to 76 kg (average body mass: 65.98 ± 4.87 kg); and their gestational weeks range from 37 to 42 (average gestational weeks: 39.28 ± 1.05). Their ages in the study group (n = 47) were 21-35 years old (mean age: 27.49 ± 3.13 years old), height was 156-171 cm (mean stature: 164.10 ± 2.75 cm), body mass was 55-77 kg (average body mass: 66.03 ± 4.89 kg) and gestational weeks were 37-42 (average gestational weeks: 39.31 ± 1.03). Comparison showed that no statistical significance exists in both comparability groups (*P* > 0.05).

### Ethical Approval:

The study was approved by the Institutional Ethics Committee of Baoding Maternal and Child Health Hospital (No. 2019035; Date: 08 January 2019), and all participants provided written informed consent.

### Inclusion criteria

- Healthy unipara undergoing natural delivery, a single birth and LOA.
- Gestational weeks range from 37 to 41, with or without six days.
- 20-35 years old.
- Those who can have a natural delivery.

### Exclusion criteria

- Those suffering from severe endocrine system diseases.
- Those who had natural delivery were converted to caesarean delivery.
- Those suffering from mental diseases.
- Those who have no normal language communication and expression abilities.
- Those who have abnormal foetal positioning or are in induced labour due to a stillbirth.

For the control group, the Doula instrument completes perinatal intervention. A trained Doula instructor was allocated one piece of Doula instrument to provide one-on-one care. The Doula instrument was first used when the uterine orifice opened for 2-3 cm. Moreover, the Doula instrument has two channels: A and B. Channel A was attached to electrode slices on a puerpera’s both hands and Channel B was attached to her lumbosacral portion. This procedure adjusted the current density based on the labour stage and the corresponding puerpera concrete conditions. The puerpera’s main complaint is that she lacks comfort and has tremors in optimal condition.

The study group underwent the following perinatal intervention: auricular acupuncture, acupoint application and the Doula instrument. First, auricular acupuncture was used when the uterine orifice opened about 3 cm. After accurate positioning of Shenmen (both sides), sympathia (both sides) and internal secretion acupoints, topical disinfection and cleansing were performed, followed by twirling of a stainless steel acupuncture needle (specification: 0.25 × 25 mm; Shanghai Taicheng Science and Technology Development Co., Ltd.). After obtaining the needling sensation, we kept the needle in place for 0.5 hour.

In this course, observing puerperal’s tolerance, uterine contraction and labour stage was counted. Second, an acupoint application was performed when the uterine orifice opened 2-3 cm. The traditional Chinese medicine application should be well prepared in advance. In a ratio of 1:1, Achyranthes bidentata was combined with rhizoma pinellinae praeparata. The mixture was then grinded into powder and run through a sifter (200 meshes). A proper amount of honey was added, transforming the mixture into a paste fabricated into medical cakes (10 × 10 × 5 mm). Finally, the medical cakes were fixed in the centre of medical rubber cloths.

Gu (both sides) and Sanyinjiao (both sides) were selected as acupuncture points for administering the medical cakes. Once they were positioned, topical disinfection and cleaning were performed. After that, medical rubber cloths attached to the medical cakes were accurately applied to the corresponding acupuncture points, changed every four hours and removed two hours after delivery. Particular attention should be paid to local skin and puerperal physiological changes during application. In severe adverse reactions, they must be removed immediately and targeted treatment measures implemented. Third, specific operations of the Doula instrument are the same as those in the control group.

### Observation targets:

Numerical Rating Scale (NRS)^5^ score comparison. The highest score obtained based on NRS is 10. The higher the score of a puerpera is, the more severe the pain will be.

Labour time comparison. The incubation and active periods of the first stage of labour and the time of the second and third stages of labour are all recorded.

Comparison of Apgar scores for the newborns. Apgar scores of one and five minutes are recorded.

Comparison of perinatal serum stress indexes for puerperae. Peripheral venous blood was collected and centrifuged before delivery and five minutes after delivery. The serum was then examined using an enzyme-linked immunosorbent assay to evaluate the following four serum stress indexes: substance P (SP), neuropeptide tyrosine (NPY), nerve growth factor (NGF) and prostaglandin E2 (PGE_2_).

### Statistical Analysis:

SPSS23.0 was used to analyse the data. All relevant measurement data with a normal distribution are expressed in (*χ̅*±*S*). An independent-sample T-test is used for intergroup comparison, whereas a paired-sample T-test is used for intra-group comparison. Enumeration data are represented as n (%), and intergroup comparison was performed using the X^2^ test. *P* < 0.05 shows that the differences are statistically significant when *α* = 0.05.

## RESULTS

The NRS score of the study group (3.31 ± 0.82) was smaller than that of the control group (7.89 ± 0.95). Intergroup differences were statistically significant (*P* < 0.05). In comparison to the control group, the incubation and active periods of the first stage of labour and the time of the second and third stages of labour are all shorter in the study group (*P* < 0.05), as shown in Table-I.

**Table-I:** Labour time comparison (*χ̅*±*S*, h).

Groups	Number of cases	Incubation period of 1^st^ stage of labour	Active period of 1^st^ stage of labour	Time of 2^nd^ stage of labour	Time of 3^rd^ stage of labour
Control	43	7.36 ± 0.53	3.97 ± 0.24	1.41 ± 0.13	0.17 ± 0.05
Study	47	5.74 ± 0.25	3.16 ± 0.12	1.14 ± 0.07	0.11 ± 0.03
*t*	—	18.800	20.512	12.411	6.971
*P*	—	0.000	0.000	0.000	0.000

The comparison of one and five minutes Apgar scores between both groups show that their differences have no statistical significance (*P* > 0.05), as shown in Table-II. Before delivery, SP, NPY, NGF and PGE_2_ expression levels are compared, and their differences are statistically significant (*P* > 0.05). SP, NPY, NGF and PGE_2_ expression levels are higher in both groups five minutes after delivery than before. Moreover, SP, NPY, NGF and PGE_2_ expression levels in the study group are lower than in the control group (*P* < 0.05; Table-III).

**Table-II:** Apgar score comparison for the newborns (*χ̅*±*S*, min).

Groups	Number of cases	1-min Apgar scores	5-min Apgar scores
Control	43	9.78 ± 0.64	9.98 ± 0.03
Study	47	9.83 ± 0.59	9.99 ± 0.05
*t*	—	0.386	1.137
*P*	—	0.701	0.259

**Table-III:** Perinatal serum stress index comparison for puerperae (*χ̅*±*S*).

Groups	Number of cases	SP (μg/mL		NPY (μg/L	

		Before delivery	5 min after delivery	Before delivery	5 min after delivery
Control	43	2.64± ± 0.34	6.86 ± 0.81^a^	179.73 ± 16.37	259.35 ± 27.43^a^
Study	47	2.68 ± 0.36	4.23 ± 0.59^a^	179.85 ± 16.42	219.16 ± 20.28^a^
*t*	—	0.541	17.712	0.035	7.949
*P*	—	0.590	0.000	0.972	0.000
Control	43	42.53 ± 5.37	60.54 ± 7.78^a^	229.86 ± 21.77	269.49 ± 31.42^a^
Study	47	43.02 ± 5.41	50.25 ± 6.11^a^	230.04 ± 21.82	239.95 ± 25.76^a^
*t*	—	0.431	7.009	0.039	4.894
*P*	—	0.668	0.000	0.969	0.000

***Note:*** P < 0.05 for intra-group comparison before delivery^1^

## DISCUSSIONS

The present study showed that the NRS score of the study group (3.31 ± 0.82) is lower than that of the control group (7.89 ± 0.95), and the intergroup differences were statistically significant. This suggests that perinatal intervention using a combination of auricular acupuncture, acupoint application and the Doula instrument effectively alleviates labour pain in women undergoing natural delivery. Additionally, our research results show that the incubation and active periods of the first stage of labour and the time of the second and third stages are shorter in the study group than in the control group. On this basis, a perinatal intervention combining auricular acupuncture, acupoint application and the Doula instrument significantly reduces labour time taken by natural delivery. Research findings show that intergroup differences in Apgar scores at one and five minutes had no statistical significance.

This demonstrates that auricular acupuncture + acupoint application + Doula instrument perinatal intervention for natural delivery has a minor influence on the health of the newborns, showing the technique’s safety benefit. Moreover, SP, NPY, NGF and PGE_2_ are all important serum indexes associated with physiological pain. Variations in expression levels of these indexes directly and objectively reflect physiological pain conditions.^6^ By monitoring such variations, we can learn about the validity of the corresponding perinatal intervention method and ensure that the pain control effects generated by such a method are satisfactory. Finally, this study shows that SP, NPY, NGF and PGE_2_ expression levels in the study group five minutes after delivery are lower than those in the control group. This means that perinatal intervention of auricular acupuncture + acupoint application + Doula instrument resulted in a relatively low pain stress level and a slight increase in serum stress indexes.

Based on neuroscience principles, the Doula instrument can address pain care requirements during puerperae delivery periods. A Doula instructor can be assigned during the delivery period to ensure that the Doula instrument is used accurately and that pain-relieving parameters are adjusted in accordance with physical truth. The application effects of the Doula instrument are expected to be enhanced in this manner.^7^-^8^ Auricular acupuncture regulates physiological activities primarily by stimulating acupuncture points distributed in the aural region for disease diagnosis and treatment.^9^-^10^ Auricular points are positions where vital energy and blood meet. We can directly communicate with zang-fu viscera, channels and organs in the human body by acting on the surface skin of the ears.^11^ In the present study, Shenmen (both sides), sympathia (both sides) and internal secretion acupoints are used to perform auricular acupuncture. Stimulating the internal secretion acupoint, in particular, may regulate physiological activities and achieve intercoordination of different zang-fu viscera.^12^

Acupoint application is an effective perinatal intervention method that integrates multiple actions, including drugs, acupoints and main and collateral channels. When implementing acupoint application, Hegu and Sanyinjiao acupoints are simultaneously stimulated, allowing such an intervention to produce dual effects of uplifting yang qi and regulating Yin and blood. Intercoordination of such two points is most suitable for pain relief.^13^ It has been reported in modern medical research that stimulating Hegu and Sanyinjiao acupoints can reinforce adrenal gland activities, increase cortisol secretion and expedite child delivery during childbirth.

As a result, the labour time and the duration of uterine contraction-inducing pain are lowered, reducing the likelihood of adverse events.^14^-^16^ A. bidentata combined with rhizoma pinellinae praeparata has the potential to benefit qi and blood, preserving certain forces of labour, meeting delivery demands and improving delivery quality.^17^ According to modern medical research findings, triterpenoid saponin, an effective constituent in A. bidentata, influences uterine smooth muscle contraction; multiple effective constituents of rhizoma pinellinae praeparata, including protein and saponin, can directly act on uterine to strengthen contractions and shorten labour time, ensuring smooth delivery.^18^

Delivery is an important step in the female fertility process. Psychological disorders are aggravated among most puerperae who suffer from intense pain due to inadequate knowledge about delivery. Specific conditions of psychological disorders inevitably directly impact the entire delivery process, such as increasing the occurrence probability of uterine inertia. It is a common risk occurrence in terms of uterine inertia. Once it occurs, the labour time must be prolonged, endangering the lives of the puerperae and foetuses.^19^ Therefore, perinatal pain intervention is required because improving the ratio occupied by natural delivery and ensuring maternal and child safety is crucial.

### Limitations:

The observation targets selected for the present study are all associated with labour analgesia. However, due to a small sample size and a single sampling source, the research achievements presented in this study may be biased. Therefore, more research is needed.

## CONCLUSIONS

Puerperae undergoing natural delivery are intervened in their perinatal period by combining auricular acupuncture, acupoint application and a Doula instrument. It turns out that such an approach can significantly reduce labour pain and shorten labour time without impairing the health status of newborns or aggravating pain stress degree in the perinatal period of puerperae.

### Authors’ contributions:

**BS** and **JR:** Designed this study, prepared this manuscript, are responsible and accountable for the accuracy and integrity of the work.

**YY** and **CY:** Collected and analysed clinical data.
